# Immersive virtual reality as a novel approach to investigate the association between adverse events and adolescent paranoid ideation

**DOI:** 10.1007/s00127-024-02701-6

**Published:** 2024-06-28

**Authors:** Charlotte Gayer-Anderson, Gemma Knowles, Stephanie Beards, Alice Turner, Daniel Stanyon, Sam Davis, Rachel Blakey, Katie Lowis, Lynsey Dorn, Aisha Ofori, Mar Rus-Calafell, Craig Morgan, Lucia Valmaggia

**Affiliations:** 1 Health Service and Population Research Department, Institute of Psychiatry, Psychology & Neuroscience, ESRC Centre for Society and Mental Health, King’s College London, London, SE5 8AF UK; 2 ESRC Centre for Society and Mental Health, King’s College London, London, UK; 3 Centre for Evidence and Implementation, London, UK; 4 School of Psychology, University of Surrey, Guildford, UK; 5 Research Center for Social Science & Medicine, Tokyo Metropolitan Institute of Medical Science, Tokyo, Japan; 6 MRC Integrative Epidemiology Unit, University of Bristol, Bristol, UK; 7 Oxford Institute of Clinical Psychology Training and Research, Oxford, UK; 8 Clinical Child and Adolescent Psychology, Ruhr-Universität Bochum, Bochum, Germany; 9 Orygen Centre for Youth Mental Health, University of Melbourne, Melbourne, Australia

**Keywords:** Adolescence, Adversity, Aetiology, Bullying, Paranoid ideation, Virtual reality

## Abstract

**Purpose:**

Paranoid ideation is common among adolescents, yet little is known about the precursors. Using a novel immersive virtual reality (VR) paradigm, we tested whether experiences of bullying, and other interpersonal/threatening events, are associated with paranoid ideation to a greater degree than other types of (i) non-interpersonal events or (ii) adverse childhood experiences.

**Methods:**

Self-reported exposure to adverse life events and bullying was collected on 481 adolescents, aged 11–15. We used mixed effects (multilevel) linear regression to estimate the magnitude of associations between risk factors and paranoid ideation, assessed by means of adolescents’ reactions to ambiguously behaving avatars in a VR school canteen, adjusting for putative confounders (gender, year group, ethnicity, free school meal status, place of birth, family mental health problems).

**Results:**

Lifetime exposure to interpersonal/threatening events, but not non-interpersonal events or adverse circumstances, was associated with higher levels of state paranoid ideation, with further evidence that the effect was cumulative (1 type: ϐ_adj_ 0.07, 95% CI -0.01-0.14; 2 types: ϐ_adj_ 0.14, 95% CI 0.05–0.24; 3 + types: ϐ_adj_ 0.24, 95% CI 0.12–0.36). More tentatively, for girls, but not boys, recent bullying was associated with heightened paranoid ideation with effect estimates ranging from ϐ_adj_ 0.06 (95% CI -0.02-0.15) for physical bullying to ϐ_adj_ 0.21 (95% CI 0.10–0.32) for cyber bullying.

**Conclusions:**

Our data suggest a degree of specificity for adversities involving interpersonal threat or hostility, i.e. those that involve unwanted interference and/or attempted control of an individual’s personal boundaries being associated with heightened levels of state paranoid ideation among adolescents.

**Supplementary Information:**

The online version contains supplementary material available at 10.1007/s00127-024-02701-6.

## Introduction

Adolescence is a critical window for the development of social cognition and the social brain [1]. Simultaneously, adolescents must navigate substantial changes in their social worlds, including the formation of hierarchical peer relationships, bullying, and other social adversities. The corollary of these changes is hypersensitivity to acceptance and rejection by peers and increased emotional lability [2]. Whilst most adolescents will be successful in navigating this transitional period, for others, normative concerns regarding others’ intentions may become negatively biased such that ambiguous behaviours of others may be perceived as hostile.

Paranoia, a multi-dimensional phenomenon involving unjustified beliefs that others have hostile intentions and may want to cause harm, may be a particular concern during adolescence. High estimates of suspiciousness and paranoid beliefs about others (~ 25%) have been reported in community samples of adolescents [3, 4]. These are often accompanied by high levels of distress, symptoms of anxiety, depression, and conduct problems [3], and an increased risk of mental health problems in adulthood, including psychotic disorders [5]. To design early and improved preventative interventions, it is crucial to more thoroughly understand the broader risk factors that may influence mental ill-health symptoms among young people.

In non-clinical adolescent samples, childhood abuse (physical, sexual, emotional) and bullying have been found to be robust risk factors in the development of paranoid thinking [6–8]. This is in line with cognitive models of paranoia which suggest that adverse childhood experiences create long-lasting negative beliefs about the self as vulnerable and the world as hostile which, in turn, manifests in emotional distress and paranoia [9]. These often-studied forms of victimisation represent acute, traumatic stressors, but they also often occur against a backdrop of chronic family adversity (e.g. marital discord, poverty, parental alcohol problems) and an array of other non-interpersonal events (e.g. serious accident, death of a parent). Further research is required to disentangle and isolate the specific impacts of acute interpersonal and/or threatening events from other stressful contexts and deepen our understanding of the aetiology of subclinical or clinical symptoms of psychosis and the potential mechanisms that underlie these associations [10, 11]. With some exceptions [12–16], relatively little attention has been given to more diverse environmental exposures, beyond those that involve overt and intentional harm, in the development of psychotic symptoms among adolescents. The limited available evidence suggests that experiences involving an “intention to harm” are more strongly associated with the report of psychotic symptoms [12–15]. Far less attention has been paid to the possible wider environmental exposures associated with the formation and maintenance of paranoid beliefs more specifically (as opposed to psychotic symptoms broadly defined).

Immersive virtual reality (VR) has been pioneered to investigate the factors associated with the onset and maintenance of paranoid appraisals in adults [17], with strong convergent, criterion, and predictive validity [18, 19]. Individuals experience and interact with computer-generated 3D social environments in a head-mounted display, and are asked to rate their level of suspiciousness towards ambiguously behaving avatars (e.g. looking at and then away from the user). The foremost benefits of using VR to assess state levels of paranoid ideation are that: (i) it affords high reliability and ecological validity, and an ability to tease out social reactions in the context of a standardised exposure; (ii) it allows for examination of real-time in-situ psychological and physiological reactions to social situations, reducing self-report/observer bias, and, (iii) individuals cannot provoke hostile reactions from the avatars, thereby facilitating interpretations relating to the direction of effect. Therefore, unlike self-report measures of paranoia that typically capture global and enduring beliefs about the negative intentions of others towards the self (i.e., trait paranoid ideation), VR captures in the moment variations in responses to and interpretations of specific situations (i.e., state paranoid ideation). The use of virtual environments that lack an objective threat to measure state paranoid ideation helps to reduce the difficulty of knowing whether attributions of harm are unfounded, and/or not too excessive given the context – i.e. what has been coined as the ‘paranoia problem’ [20].

This paper outlines the use of a novel immersive VR paradigm, the first (to our knowledge) to be age appropriate for use in adolescents, to assess state paranoid ideation. In a community sample of adolescents, we sought to test the hypotheses that experiences of bullying, and other interpersonal and/or threatening events (e.g. being physically hit or hurt), are associated with unjustified paranoid ideation towards ambiguously behaving avatars within a VR environment to a greater degree than (i) non-interpersonal events (e.g. experiencing a serious accident or illness) or (ii) other adverse childhood experiences (e.g. frequent parental discord). Given higher rates of paranoia in adolescent girls [4, 21], and gender differences in the experience and psychological expressions of bullying [22], post-hoc analyses were carried out to examine whether the association between interpersonal and/or threatening events and paranoid ideation would be more pronounced in girls.

## Methods

### Participants

A sub-sample of adolescents was recruited between 2017 and 2019 from the Resilience and Ethnicity in AdolesCent Mental Health study (REACH) [23], an accelerated cohort study of adolescent mental health in the two inner-city London boroughs. Seven of the 12 schools involved in REACH agreed to take part in the VR sub-study. After completion of the REACH main assessments, students and their carers were provided with information about the VR sub-study. Written informed consent was received from carers/guardians of 527 students. Among those consented, reasons for not participating were: being absent from school/unavailable at the time of assessment (n 42, 8.0%), cyber-sickness whilst using the VR headset (n 3, 0.6%), and technical difficulties resulting in invalid results (n 1, 0.2%). Written informed assent was obtained from all students prior to assessment. All procedures were approved the Psychiatry, Nursing and Midwifery Research Ethics Subcommittee (PNM RESC), King’s College London (ref: 15/162,320).

### Virtual reality

**Fig. 1:**
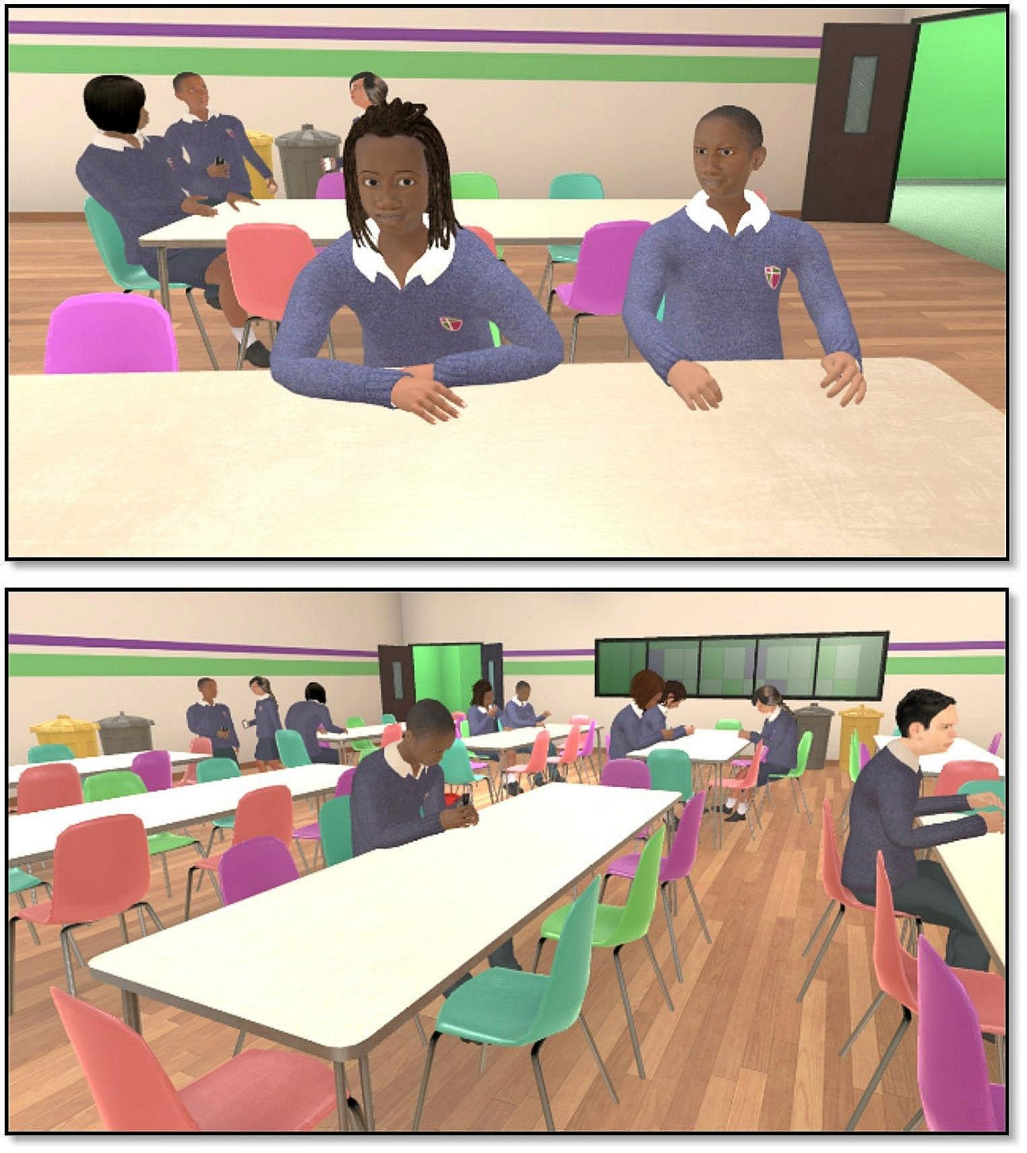
Screenshots of the VR canteen environment. The school children were designed to look between the ages of 12–14 years old, and represented various ethnicities (mostly black African/Caribbean, in order to simulate the ethnic composition of schools participating in REACH). For a video please see https://www.youtube.com/watch?v=x-ZzkhgWcEM.

The virtual environment, designed by the KCL Institute of Psychiatry, Psychology and Neuroscience VR Lab and built by Virtualware, is a school canteen, populated by computer characters (‘avatars’) of 22 school children and a member of canteen staff (see Fig. 1 and S1. Supplementary Methods for more detail). Once in the canteen, participants were provided with a series of instructions, each one directing the participant to approach the avatars. There were four brief interactions, each designed to be ambiguous in how they could be perceived and interpreted. Whilst the scenario involves a standardised narrative (i.e. all participants ‘walk’ around the canteen on the same predefined route, and all avatars behave in exactly the same way for all participants), some of these interactions were designed to encourage the participants to explicitly engage with the avatars (e.g. by verbally responding to questions posed by the avatars, or waving hello), thereby to enhance ecological validity of the paradigm.

### Pre-VR measures

Basic demographic information, and exposure to bullying and lifetime events and difficulties, was collected as part of REACH, during in-class computerised battery of questionnaires on study tablets.

#### Bullying

The Bully, Victim Questionnaire [24] asks students to indicate how often each type of bullying (physical, verbal, neglect, and cyber) was experienced in the last six months (S1 Supplementary Methods). Students answered on a five-point scale from never to several times a week. For analyses, responses were dichotomised to reflect exposure to any frequency of bullying versus none for each type of bullying. A summary count of the number of types of bullying reported (0–4) was also calculated.

#### Discrimination

Questions were asked on lifetime experiences of discrimination about race, skin colour, or place of birth [25]. A single dichotomous variable was constructed with any positive responses coded as the ‘yes’ category.

#### Life events

An amended version of the adolescent-appropriate Life Events Checklist [26] was used to collect information on lifetime exposure to 24 difficult events. In line with previous research [27], three summary count variables were created to elicit lifetime exposure to (i) Interpersonal Events (5 items including the question on discrimination, see above), (ii) Non-Interpersonal Events (13 items), and (iii) Adverse Childhood Circumstances (6 items). These variables were subsequently recoded into an ordinal variable to represent report of exposure to 0, 1, 2, and 3 + types of events in each category.

#### Putative confounders

Participants were asked to describe their ethnicity from a list of 18 categories used in the 2011 Office for National Statistics census. Due to low numbers in some groups (e.g. Chinese, Arab), we combined smaller groups resulting in eight ethnic groups: white British; other white; Latin American; black African; black Caribbean; other black; Asian; mixed/multiple ethnicities. Free school meal status (yes/no), a marker of socioeconomic status based on household income, was collected via a single self-report item. Place of birth and gender were also self-reported. A dichotomous variable for any family (parent/sibling) mental health problems was created based on students’ responses (yes/no) to a checklist of eight mental health problems. A positive response to any of these items equated to family mental health problems. See S1 Supplementary Methods for details on the specific questions asked.

### VR-specific measures

#### State paranoid ideation

Paranoid ideation about the VR experience was measured using a shortened version of the State Social Paranoia Scale (SSPS) [28]. This comprises eight items (S1. Supplementary Methods) to assess levels of paranoid (5 items), neutral (1 item) and positive (2 items) thoughts towards the avatars, each rated on a five-point scale from “do not agree” to “totally agree”. Higher scores on the five paranoia items combined indicate greater levels of paranoid thinking (possible range 5 to 25). The scale was administered after the participants completed the VR social scenario, within the headset. The full version of the SSPS has excellent internal reliability (α = 0.91), good test-retest reliability (*r* = 0.73), and clear convergent validity as assessed by both independent interviewer and self-report ratings [28].

#### Immersion in the VR experience

A visual analogue scale (VAS) was used to assess presence (i.e., how real the environment felt), scored from 0 (feel as though you are doing research in a school classroom) to 100 (feel as though you are in a virtual canteen) [29].

#### Convergent/divergent validity

Additional VAS ratings were collected to assess “How paranoid did you feel in the school canteen” [19], and how hostile, neutral, or friendly the avatars were (scored from 0 [low] to 100 [high]).

#### Frequency of gaming

Frequency of playing video games was assessed with a single item rated on a six-point scale (daily to never).

### Analysis

Mixed effects (multilevel) linear regression models were used to estimate magnitude of associations between putative risk factors (bullying types (independent effects and cumulative), and the cumulative effects of (i) interpersonal events, (ii) non-interpersonal events, and (iii) adverse childhood circumstances) and paranoid ideation. To account for clustering of participants within schools, school was fitted as a second-level variable in all models. We examined variations in effect by gender, by fitting interaction terms to the models and conducting likelihood ratio tests to assess whether the interaction terms improved model fit. The residuals in these models were non-normally distributed, so log-transformed paranoia scores were used. For interpretation, log means are presented as back-transformed (i.e. exponentiated). Analyses were adjusted for putative confounders, including gender, year group, ethnicity, free school meal status, and place of birth. Due to the extent of missing data on family mental health history (35% missing), separate complete case analyses were conducted to determine whether family mental health was a potential confounder. Analyses were performed in Stata 15.

## Results

In total, 481 students (56% girls) were assessed in the VR canteen paradigm. Most were from non-white British ethnic background (63%) and 11% were born outside of the UK (Table 1). Around 16% reported receiving free school meals.

**Table 1:** Associations between demographic variables and levels of VR paranoid ideation scores (log-transformed)

			VR paranoia scores		Unadjusted			Unadjusted*		
	*n*	%	Mean	s.d.	ϐ	95% CI	*p*	ϐ	95% CI	*p*
**Gender**										
Boys	210	43.7	9.10	3.54	-			-		
Girls	271	56.3	9.49	3.41	0.05	-0.01; 0.11	0.126	0.07	0.01; 0.14	0.049
**Year group**										
7 (age 11–12)	92	19.1	8.70	2.70	-			-		
8–9 (age 12–14)	281	58.4	9.70	3.77	0.08	0.00; 0.17	0.045	0.14	0.03; 0.24	0.009
10–11 (age 14–16)	108	22.5	8.85	3.11	0.01	-0.09; 0.10	0.944	0.11	-0.01; 0.23	0.082
**Ethnicity** ^**a**^										
White British	175	36.5	9.81	3.30	-			-		
Other White	52	10.9	10.04	3.80	0.01	-0.09; 0.12	0.830	0.01	-0.09; 0.11	0.843
Latin American	13	2.7	12.23	5.45	0.18	-0.01; 0.37	0.070	0.25	0.06; 0.44	0.011
Asian	43	9.0	8.21	2.49	-0.17	-0.28; -0.05	0.004	-0.08	-0.20; 0.04	0.204
Black African	79	16.5	8.49	3.24	-0.16	-0.25; -0.06	0.001	-0.08	-0.18; 0.02	0.114
Black Caribbean	33	6.9	7.97	3.09	-0.21	-0.34; -0.08	0.001	-0.14	-0.27; -0.01	0.041
Other black	15	3.1	8.60	2.92	-0.14	-0.32; 0.04	0.131	-0.09	-0.26; 0.09	0.343
Mixed	69	14.4	9.36	3.50	-0.06	-0.15; 0.04	0.221	-0.03	-0.12; 0.06	0.515
**Child birth place** ^**b**^										
UK	426	89.1	9.23	3.38	-			-		
Outside UK	52	10.9	9.88	4.01	0.06	-0.04; 0.16	0.262	0.10	-0.01; 0.19	0.055
**Eligible for free school meals** ^**c**^										
No	385	84.4	9.44	3.35	-			-		
Yes	71	15.6	8.76	3.98	-0.10	-0.18; -0.01	0.032	-0.06	-0.14; 0.03	0.208
**Mental health problems in parents / siblings** ^**d**^										
No	191	58.8	8.71	3.25	-			-		
Yes	134	41.2	10.07	3.94	0.14	0.06; 0.22	< 0.001	0.12	0.04; 0.20	0.003
**Level of immersion in the canteen** ^**e**^										
0–49 (low)	118	27.9	9.28	3.32	-			-		
50–100 (high)	305	72.1	9.60	3.55	0.03	-0.04; 0.11	0.399	0.04	-0.03; 0.11	0.308
**Frequency of gaming** ^**f**^										
Very infrequently	151	33.9	9.60	3.37	-			-		
1-2x a month	78	17.5	9.10	3.43	-0.05	-0.15; 0.04	0.283	-0.04	-0.13; 0.05	0.400
Weekly, daily	217	48.6	9.34	3.58	-0.03	-0.10; 0.04	0.402	-0.06	-0.13; 0.02	0.138

ϐ, unstandardised linear regression coefficient; CI, confidence interval; *accounting for clustering by school; Missing data from: ^a^2 students; ^b^3 students; ^c^25 students; ^d^156 students; ^e^58 students; ^f^36 students

### Convergent validity and immersion

Mean VAS scores for the perceived neutrality and hostility of the computer avatars were comparable (neutral mean VAS score 40.0, s.d. 25.5; hostile mean VAS score 40.0, s.d. 28.6). Lower scores were endorsed for friendliness of the avatars (mean VAS score 20.0, s.d. 24.2). SSPS paranoid ideation scores ranged from 5 to 23 and were positively skewed (Supplementary Fig. S2), and the items demonstrated acceptable internal consistency (α = 0.73). Positive/neutral items were commonly endorsed with 82% (n 394) reporting some positive or neutral thoughts about the avatars. There was a modest correlation (r_s_=0.367, *p* < 0.001) between VR paranoid ideation scores and the post-VR VAS which asked the individual how paranoid they felt generally within the canteen. Sense of presence VAS scores revealed a high level of immersion; the majority (72%, n 306) of the participants scoring 50 and above.

### Demographic characteristics

Levels of paranoid ideation were higher among girls (ϐ 0.07, 95% CI 0.01–0.14), and in students born outside the UK (ϐ 0.10, 95% CI -0.01-0.19) (Table 1). Compared with students in Year 7 (ages 11–12), older students reported higher levels of paranoid ideation (Years 8–9, ages 12–14: ϐ 0.14, 95% CI 0.03–0.24; Years 10–11, ages 14–16: ϐ 0.11, 95% CI -0.01-0.23). We found some variation by ethnic group; compared with white British students, those in the Latin American group reported higher levels of paranoid ideation (ϐ 0.25, 95% CI 0.06–0.44), whilst individuals from black Caribbean (ϐ -0.14, 95% CI -0.27- -0.01), and to a lesser extent black African origins (ϐ 0.08, 95% CI -0.18-0.02) reported lower paranoid ideation. In addition, report of mental health problems within family members was associated with higher levels of paranoid ideation (ϐ 0.12, 95% CI 0.04–0.20).

### Bullying

Around 60% of the sample reported exposure to at least one form of bullying in the previous six months. Exposure to each type of bullying in the previous six months was associated with higher levels of paranoid thoughts, independent of gender, year group, ethnicity, place of birth, and free school meal status (**Table 2**). The magnitude of the effects ranged from 0.05 (95% CI -0.02-0.11; physical bullying) to 0.15 (95% CI 0.06–0.24; cyber bullying). Crucially, as the number of types of reported bullying increased, so did the levels of paranoid ideation. Those who reported three types of bullying scored on average an increase of 0.11 (95% CI 0.01–0.22) in levels of paranoia than those who reported no bullying, which increased to 0.28 (95% CI 0.12–0.45) for those who reported all four types of bullying. There was no evidence that a non-linear relationship between cumulative types of bullying and level of paranoia provided a better fit for the data (χ = 0.76, *p* = 0.383). No attenuation in the effect sizes was found when repeating the analyses on those with complete data on the additional putative confounder of family history of mental health problems (Supplementary Table S5).

#### By gender

When the sample was stratified by gender (Table 3), formal tests of interaction were indicative of differences in the effects on paranoid ideation between girls and boys for all types of bullying (apart from physical), and the cumulative effects of bullying. For boys, there was little evidence that any form of bullying was independently associated with paranoid ideation, with coefficients ranging between − 0.01 (for verbal bullying, 95% CI -0.12-0.09) and 0.04 (for those ostracised by peers, 95% CI -0.07-0.14). For girls, all forms of bullying were associated with higher levels of paranoid ideation, with ϐ_adj_ coefficients ranging between 0.06 (for physical bullying, 95% CI --0.02-0.015) and 0.21 (for cyber bullying, 95% CI 0.10–0.32).

**Table 2:** Associations between bullying in the previous six months, and lifetime events and difficulties, and levels of VR paranoid ideation scores (log-transformed)

			VR paranoia scores		Unadjusted			Unadjusted *			Adjusted *†			Adjusted *‡			
	*n*	%	mean	s.d.	ϐ	95% CI	*p*	ϐ	95% CI	*p*	ϐ	95% CI	*p*	ϐ	95% CI	*p*	
**Physical bullying** ^**a**^																	
No	232	51.0	8.95	3.21	-			-			-			-			
Yes	223	49.0	9.55	3.52	0.06	-0.01; 0.13	0.051	0.04	-0.03; 0.10	0.265	0.04	-0.02; 0.10	0.198	0.05	-0.02; 0.11	0.139	
**Verbal bullying** ^**b**^																	
No	312	68.3	9.01	3.30	-			-			-			-			
Yes	145	31.7	9.93	3.65	0.10	0.03; 0.17	0.004	0.07	-0.01; 0.13	0•051	0.07	0.01; 0.14	0.034	0.08	0.01; 0.15	0.031	
**Neglect bullying** ^**c**^																	
No	381	82.8	9.14	3.22	-			-			-			-			
Yes	79	17.2	9.94	4.09	0.07	-0.02; 0.15	0.111	0.04	-0.04; 0.13	0.290	0.07	-0.01; 0.16	0.077	0.09	0.01; 0.18	0.028	
**Cyber bullying** ^**d**^																	
No	400	86.8	9.07	3.30	-			-			-			-			
Yes	61	13.2	10.84	3.93	0.17	0.08; 0.27	< 0.001	0.15	0.06; 0.24	0.001	0.14	0.05; 0.23	0.003	0.15	0.06; 0.24	0.001	
**Cumulative experiences of bullying** ^**e**^																	
None	182	39.7	8.76	3.26	-			-			-			-			
One type	134	29.2	9.50	3.54	0.08	0.01; 0.16	0.035	0.05	-0.02; 0.13	0.162	0.05	-0.03; 0.12	0.197	0.07	-0.01; 0.14	0.070	
Two types	72	15.7	9.11	2.68	0.06	-0.03; 0.15	0.214	0.02	-0.07; 0.11	0.638	0.03	-0.06; 0.13	0.474	0.03	-0.06; 0.13	0.469	
Three types	54	11.8	9.96	3.41	0.14	0.03; 0.24	0.011	0.10	-0.01; 0.20	0.056	0.11	0.01; 0.21	0.041	0.11	0.01; 0.22	0.033	
Four types	17	3.7	12.00	5.63	0.27	0.10; 0.44	0.002	0.22	0.05; 0.38	0.011	0.24	0.07; 0.40	0.005	0.28	0.12; 0.45	0.001	
**Interpersonal events** ^**f**^																	
0 types of events	153	33.6	8.65	2.81	-			-			-			-			
1 types of events	185	40.6	9.13	3.46	0.04	-0.04; 0.11	0.316	0.05	-0.03; 0.12	0.204	0.05	-0.02; 0.12	0.176	0.07	-0.01; 0.14	0.077	
2 types of events	75	16.4	9.83	3.51	0.12	0.03; 0.22	0.013	0.12	0.03; 0.21	0.009	0.13	0.04; 0.22	0.006	0.14	0.05; 0.24	0.003	
3 + types of events	43	9.4	10.93	4.57	0.20	0.08; 0.32	< 0.001	0.20	0.09; 0.31	< 0.001	0.20	0.09; 0.32	< 0.001	0.24	0.12; 0.36	< 0.001	
**Non-interpersonal events** ^**g**^																	
0 types of events	27	5.8	9.26	2.75	-			-			-			-			
1 types of events	87	18.6	8.68	3.09	-0.07	-0.23; 0.08	0.332	-0.06	-0.20; 0.09	0.448	-0.06	-0.21; 0.08	0.384	-0.08	-0.23; 0.06	0.264	
2 types of events	86	18.3	9.02	2.71	-0.03	-0.18; 0.13	0.743	-0.01	-0.15; 0.14	0.898	-0.03	-0.17; 0.12	0.719	-0.03	-0.17; 0.12	0.710	
3 + types of events	269	57.4	9.57	3.81	0.01	-0.13; 0.14	0.923	0.04	-0.09; 0.18	0.521	0.03	-0.11; 0.16	0.706	0.02	-0.12; 0.16	0.769	
**Adverse childhood circumstances** ^**h**^																	
0 types of events	184	40.5	9.02	3.28	-			-			-			-			
1 types of events	137	30.2	9.37	3.66	0.03	-0.05; 0.11	0.420	0.03	-0.04; 0.11	0.387	0.02	-0.05; 0.10	0.589	0.03	-0.04; 0.11	0.383	
2 types of events	81	17.8	9.59	3.75	0.06	-0.04; 0.15	0.237	0.07	-0.02; 0.16	0.130	0.05	-0.04; 0.14	0.286	0.06	-0.03; 0.15	0.169	
3 + types of events	52	11.5	9.60	3.27	0.07	-0.04; 0.18	0.199	0.07	-0.04; 0.17	0.217	0.06	-0.05; 0.16	0.294	0.01	-0.10; 0.12	0.876	

ϐ, unstandardised linear regression coefficient; CI, confidence interval; *accounting for clustering by school; †adjusted for gender, year group; ‡ adjusted for gender, year group, ethnicity, birth place, free school meal status; Missing data for: ^a^26 students; ^b^24 students; ^c^21 students; ^d^20 students; ^e^22 students; ^f^25 students; ^g^12 students; ^h^27 students;

**Table 3:** Associations between bullying and levels of log-transformed VR paranoid ideation scores, by gender

	BOYS								GIRLS							
				VR paranoia scores		Adjusted *‡					VR paranoia scores		Adjusted *‡			Test of interaction
	n		%	Mean	s.d.	ϐ	95% CI	*p*	n	%	mean	s.d.	ϐ	95% CI	*p*	
**Physical bullying**																
No	87		44…9	8.89	3.25	-			145	55.6	8.99	3.19	-			
Yes	107		55.1	9.18	3.55	0.02	-0.07; 0.12	0.608	116	44.4	9.89	3.48	0.06	-0.02; 0.15	0.128	χ 0.38, p 0.536
**Verbal bullying**																
No	139		70.9	9.11	3.56	-			173	66.3	8.92	3.09	-			
Yes	57		29.1	9.21	3.50	-0.01	-0.12; 0.09	0.812	88	33.7	10.40	3.68	0.13	0.05; 0.22	0.003	χ 4.43, p 0.035
**Neglect bullying**																
No	143		73.3	9.00	3.42	-			238	89.8	9.23	3.09	-			
Yes	52		26.7	9.15	3.40	0.04	-0.07; 0.14	0.506	27	10.2	11.44	4.89	0.19	0.05; 0.32	0.007	χ 2.89, p 0.089
**Cyber bullying**																
No	177		90.3	9.05	3.48	-			223	84.2	9.08	3.16	-			
Yes	19		9.7	9.47	4.01	0.03	-0.13; 0.19	0.711	42	15.8	11.45	3.78	0.21	0.10; 0.32	< 0.001	χ 3.28, p 0.070
**Cumulative experiences of bullying**																
None	72		36.7	8.86	3.34	-			110	41.8	8.69	3.22	-			
One type	55		28.1	9.69	4.19	0.08	-0.04; 0.19	0.195	79	30.0	9.37	3.02	0.06	-0.03; 0.16	0.200	
Two types	36		18.4	8.47	2.68	-0.08	-0.22; 0.06	0.262	36	13.7	9.75	2.56	0.13	0.01; 0.25	0.041	
Three types	24		12.2	9.17	3.34	0.06	-0.10; 0.21	0.472	30	11.4	10.60	3.39	0.16	0.02; 0.30	0.025	
Four types	9		4.6	10.00	4.36	0.10	-0.14; 0.33	0.421	8	3.0	14.25	6.32	0.45	0.22; 0.68	< 0.001	χ 9.84, p 0.043

ϐ, unstandardised linear regression coefficient; CI, confidence interval; *accounting for clustering by school; ‡adjusted for year group, ethnicity, birth place, free school meal status

**Table 4:** Associations between lifetime events and difficulties, and levels of log-transformed VR paranoid ideation scores, by gender

	BOYS								GIRLS							
				VR paranoia scores		Adjusted *‡					VR paranoia scores		Adjusted *‡			Test of interaction
	n		%	Mean	s.d.	ϐ	95% CI	*P*	n	%	Mean	s.d.	ϐ	95% CI	*P*	
**Interpersonal events**																
0 types of events	56		28.7	8.34	2.82	-			97	37.2	8.82	2.80	-			χ 1.12, p 0.773
1 types of events	75		38.5	9.03	3.55	0.10	-0.02; 0.21	0.104	110	42.2	9.20	3.41	0.04	-0.05; 0.14	0.336	
2 types of events	40		20.5	9.23	3.33	0.13	-0.01; 0.26	0.068	35	13.4	10.51	3.62	0.16	0.04; 0.29	0.012	
3 + types of events	24		12.3	10.88	4.91	0.26	0.10; 0.42	0.002	19	7.3	11.00	4.24	0.21	0.05; 0.38	0.011	
**Non-interpersonal events**																
0 types of events	12		5.9	9.92	3.50	-			15	5.6	8.73	1.94	-			χ 4.04, p 0.257
1 types of events	38		18.8	7.68	2.17	-0.20	-0.42; 0.01	0.067	49	18.4	9.45	3.48	0.01	-0.18; 0.21	0.883	
2 types of events	30		14.9	8.47	2.66	-0.13	-0.37; 0.10	0.260	56	21.0	9.32	2.70	0.04	-0.15; 0.23	0.668	
3 + types of events	122		60.4	9.60	3.94	-0.02	-0.22; 0.18	0.821	147	55.1	9.55	3.71	0.06	-0.13; 0.24	0.545	
**Adverse circumstances**																
0 types of events	89		45.6	8.74	3.33	-			95	36.7	9.28	3.23	-			χ 4.33, p 0.228
1 types of events	53		27.2	9.83	4.28	0.11	-0.01; 0.23	0.059	84	32.4	9.08	3.20	-0.02	-0.12; 0.08	0.694	
2 types of events	27		13.9	9.78	3.85	0.10	-0.05; 0.25	0.183	54	20.9	9.50	3.73	0.04	-0.08; 0.15	0.509	
3 + types of events	26		13.3	8.69	2.26	-0.02	-0.18; 0.13	0.763	26	10.0	10.50	3.87	0.05	-0.11; 0.20	0.552	

ϐ, unstandardised linear regression coefficient; CI, confidence interval; *accounting for clustering by school; ‡adjusted for year group, ethnicity, birth place, free school meal status

### Lifetime adverse events

Associations between the individual types of lifetime events and level of paranoid ideation are shown in Supplementary Table S6. When grouped according to the different categories (i.e., interpersonal and threatening events, non-interpersonal events, adverse circumstances), a linear effect was found for the association between interpersonal events and paranoid ideation (1 type: ϐ_adj_ 0.07, 95% CI -0.01-0.14; 2 types: ϐ_adj_ 0.14, 95% CI 0.05–0.24; 3 + types: ϐ_adj_ 0.24, 95% CI 0.12–0.36) (**Table 2**). There was no evidence of heightened paranoid ideation among adolescents exposed to non-interpersonal events, nor to adverse circumstances. Family history of mental health problems did not confound the association between lifetime events/difficulties and paranoid ideation (Supplementary Table S7).

#### By gender

For both boys and girls, cumulative exposure to interpersonal events was associated with progressively higher levels of paranoid ideation, with little difference in the effect sizes at each level of exposure (χ = 1.12, *p* = 0.773) (**Table 4**). For non-interpersonal events and adverse circumstances, there were tentative indications of differences by gender (though not supported by tests for interaction: χ = 4.04, *p* = 0.257, and χ = 4.33, *p* = 0.228, respectively). For girls, there was a trend for paranoia to increase as number of reported non-interpersonal events, and adverse circumstances, increased. By contrast, for example, we found lower levels of paranoia in boys who had reported one or two types of non-interpersonal event compared to those who reported none (1 event: ϐ_adj_ -0.20, 95% CI -0.42-0.01; 2 events: ϐ_adj_ -0.13, 95% CI -0.37-0.10).

## Discussion

To our knowledge, this is the first study to examine associations between different types of adversity and paranoid ideation in a sample of adolescents using an age-appropriate VR experimental paradigm. Several notable findings emerged. Bullying and other interpersonal/threatening events (e.g. being physically hit or hurt, victim of a mugging), but not non-interpersonal events, nor adverse circumstances, were associated with higher levels of state paranoid ideation, with further evidence that the effect was cumulative. These associations remained robust when accounting for putative confounders, including family history as proxy genetic risk. Moreover, we found some evidence of variation by gender; reported exposure to recent bullying was most strongly associated with heightened paranoid ideation in girls.

### Methodological considerations

Some methodological limitations need to be highlighted. First, this is the first time this novel VR scenario has been used to assess paranoid beliefs among adolescents. Validation of the scenario was a supplementary objective of this study. We found evidence in support of its validity, in that the scenario elicited high levels of feelings of presence in the environment, and was effective in producing variability in the outcome measure, assessed by means of a shortened version of the SSPS (used for brevity given the extensive battery of assessments administered to young people participating in the REACH study) [28]. It is also acknowledged that the correlation between VR paranoia and the post-VR VAS for paranoia was moderate, though similar effect sizes have been reported for studies assessing the validity of VR paradigms to assess paranoid ideation in adults [18, 30]. To further validate the school canteen scenario, in future studies it would be valuable to employ in-situ and real-time behavioural and physiological measures during the VR task which have been previously used to assess an individual’s emotional arousal, vulnerability to stress, and psychosis liability [31–33] – e.g. eye-gaze, heart-rate, and skin conductance etc.

As is typical of dimensional scales of psychotic symptoms [34], we found a skewed distribution of paranoid ideation scores elicited by the paradigm. This suggests that most individuals subscribe to low levels of paranoia, but that a substantial minority will report more intense paranoid experiences. These low scores mean that the effects described here primarily relate to mild paranoid attributions; any group differences according to the level of exposure to adverse events are relatively modest absolute increases, and still only represent mild levels of paranoid ideation. Sensitivity analyses (presented in Supplementary Table S8) suggest that comparable inferences can be made when state paranoia scores are dichotomised using the highest quartile as the threshold (i.e., representing symptoms which may be more clinically meaningful).

Third, the scenario involved a single presentation of a virtual school canteen; additional VR scenarios emulating naturalistic settings would provide more evidence for the generalisability of these results.

Fourth, although the VR method allows some inference in relation to causality (in that the VR avatars’ behaviours are standardised, and participants cannot provoke hostile reactions), the study, being cross-sectional, does not negate the possibility that baseline state levels of paranoia will increase the likelihood of exposure to bullying or other stressful life events and difficulties. It is also plausible that self-reported bullying may have also been confounded by levels of paranoia, as a person who is paranoid inherently makes unfounded attributions of harm from others. Longitudinal designs are necessary to fully appreciate the bidirectional relationship between early adversity and paranoid ideation.

Fifth, the use of self-reported exposure to bullying and other life events may have introduced bias, though such methods have been found to be acceptable [35]. Moreover, the prevalence of bullying in our sample is higher (60%) than what has been found in national samples (e.g. 30%) [36]. This is due to dichotomising the data into ‘never’ versus ‘any frequency of bullying’, when most often bullying is rated as present when it occurs at least twice a month. We took this decision because there appeared to be no difference in the effect estimates of bullying on paranoia when frequency was ‘once or twice’ versus when frequency was ‘at least twice a month’ (Supplementary Table S4), and further, by combining these two groups, this enabled us to consider, with more precision, the effects of the individual types of bullying experiences, overall and by gender. Future research would benefit from collecting corroborating reports of bullying from parents/teachers, and consideration of the severity of experiences.

Finally, although we tested a large sample, schools were recruited by convenience. As a result, our sample is not fully representative of the underlying adolescent population (Supplementary Table S3).

### Main effects

The above limitations notwithstanding, our findings, in line with our hypotheses, suggest strong evidence of an association between interpersonal events that involve a degree of threat, hostility, and/or violence, and an increase in state levels of paranoid ideation, in social situations, in a sample of inner-city resident adolescents.

Exposure to bullying has been found to be associated with paranoid thinking in adolescent non-clinical [4, 6, 7, 21] and clinical [37, 38] samples. Our findings concur with previous research and go further by: (i) exploring the individual and cumulative effects of different types of bullying; and (ii) consider whether the associations are similar in girls and boys. We found that cyber bullying had the strongest association with paranoid ideation; this is in line with other studies which have shown cyber-victims to report more social difficulties and higher anxiety and depression than those exposed to traditional forms of bullying [39]. Concerns exist that the intensity and impact of cyber bullying may be greater than traditional forms of bullying due to some unique features that set it apart [39]; for example, the limited likelihood for intervention, and its constant pervasiveness in terms of time and setting.

We also found that other lifetime events involving an element of interpersonal threat showed stronger associations with paranoid ideation than did non-interpersonal events or adverse circumstances. This resonates with an increasing body of research that has found more severe forms of adversity that involve direct threat, hostility, and violence, are particularly pertinent in the emergence of psychotic disorders [40], and report of psychotic-like experiences in non-clinical samples [12]. However, findings are equivocal in research on stressful life events and psychotic-like experiences among adolescents. For example, Vargas and colleagues reported that exposures relating to deprivation (e.g. indicators that the individuals’ environments lacked socioeconomic, material or educational resources) were associated most strongly with psychotic like experiences in adolescents, compared with discrepancy exposures (ethnic density, income inequality, social fragmentation), and objective stimulation factors (e.g. population density and exposure to crime) [16]. Indeed, in our sample, in view of the associations with individual life experiences (Supplementary Table S6), we found modest associations between paranoid ideation and the child having a serious illness, injury, or operation, or a parent or sibling experiencing a serious accident. Therefore, we cannot discount the potential risks conferred by other forms of trauma that are not interpersonal in nature.

Evidence for cumulative effects of bullying and other threatening interpersonal events is also in line with findings in previous studies of socio-environmental stressors and psychotic symptoms among adolescents [13] and disorders among adults [40]. However, there is some debate as to whether interpersonal events with an intention to harm exert their effects on psychotic experiences by means of an increased ‘exposure dose’ (given that they tend to take place over an extended period of time) in contrast to non-interpersonal events (e.g. accidents, disasters), which tend to be discrete incidents, with less likelihood of re-occurrence [13]. Grouping life events as we have (into interpersonal, non-interpersonal, and adverse circumstances) somewhat helps to clarify this issue. We found only associations between paranoid ideation and interpersonal events, but not with the other groupings of life events - one which tends to implicate discrete events (non-interpersonal events), the other chronic (adverse circumstances). It would appear then, at least for paranoid ideation, that there is some specificity to the nature of the event being interpersonal, rather than a differential association according to exposure ‘dose’ versus exposure ‘type’.

### Gender differences

In keeping with other studies of non-clinical adolescent samples [4, 7], girls exhibited higher levels of paranoid ideation than boys. Moreover, we found that the effect of bullying victimisation on levels of paranoid ideation in the VR paradigm was greater in girls than boys, with the exception of physical bullying. Though the moderating effects of gender between bullying and paranoid ideation in adolescents has largely been untested, the results here broadly echo previous research; girls tend to be more sensitive to social exclusion [41], with a higher susceptibility to negative appraisals, thus increasing their risk of paranoid thinking styles in relation to social status [7]. By contrast, we found no differences in the cumulative effects of interpersonal events on paranoid ideation in girls and boys.

### Potential mechanisms

This study suggests a link between recurrent threatening interpersonal adversities and state paranoid ideation in an adolescent sample. Our findings are consistent with prominent theories that an accumulation of early adverse experiences create an enduring cognitive vulnerability, characterised by negative schematic models of the self/others [42]. Such beliefs of the self as vulnerable, and appraisals of others as dangerous or untrustworthy, may create a tendency to attribute experiences as externally caused, perceive their social worlds as hostile, and, in turn, facilitate the formation of paranoid ideation [42]. Complementary to this, repeated and threatening interpersonal experiences in childhood may activate changes in the interconnected biological systems (e.g. hypothalamic-pituitary-adrenal axis activation), which have been implicated in the development of future mental health problems, including psychosis. We will explore some of these potential mechanisms in future papers. Finally, we must also consider the interplay between environmental risk factors and genetic influences in the development of paranoia; evidence exists of gene-environment correlations [21, 43] - where some individuals appear to have an underlying genetic propensity which jointly increases an individual’s vulnerability to being exposed to some forms of adversities, including bullying, as well as to feeling paranoid. Other studies are more suggestive of gene-environment interactions [44] – whereby those with low genetic risk for psychotic symptoms developed paranoia when exposure to environmental risk factors was high.

#### Implications

Paranoid ideation at this vulnerable young age brings in its wake not only an increased risk of a range of mental health problems in adulthood, including psychotic disorders [5], but also the potential for a compromised trajectory of cognitive, emotional, and social development and significant functional impairment [4, 8, 45]. The significance of these negatively impacting the healthy developmental process cannot be overstated. The identification of unfounded ideas of harm and mistrust of others at this age presents an opportunity for early intervention efforts to potentially reduce the risk of adolescents missing out on the positive and constructive learning experiences that they need to become fully functioning adults. The results from this study may suggest that professionals who work with children and adolescents may consider evaluating young people who have experienced an accumulation of threatening interpersonal life experiences for possible paranoid thinking, using methods that are able to identify the phenomenon, to facilitate a timely and targeted intervention. This may help to reduce the risk of these transient experiences further developing into impairing and distressing symptoms of psychosis or other symptoms of mental ill health.

## Electronic supplementary material

Below is the link to the electronic supplementary material.

Supplementary Material 1
