# Supplementary material for: Immersive virtual reality as a novel approach to investigate the association between adverse events and adolescent paranoid ideation
Source: Soc Psychiatry Psychiatr Epidemiol. 2024 Jun 28;60(2):305–18. doi: 10.1007/s00127-024-02701-6 (PMC11839835; doi:10.1007/s00127-024-02701-6)
Supplement: Supplementary file 1 — Supplementary Material 1 [file 127_2024_2701_MOESM1_ESM.docx]

**SUPPLEMENTARY MATERIALS**

**S1. Supplementary Methods**

***Description of the Virtual Reality (VR) paradigm:*** This VR paradigm involves the administration of relatively short (~4 minutes in duration), standardised (i.e. non-personalised) scenario to ensure uniformity across participants. Whilst in the virtual environment, participants move on the same predefined route around the canteen using an Xbox control pad and by physically turning their body direction.

Participants stood initially in a school corridor, and were given the instruction “You have just finished your maths lesson and are heading to the canteen for the afternoon break time. Please join the other students and try to make an impression of what you think about them and what they think about you”. These instructions are meant to encourage the participant to make some form of social evaluation towards the avatars, whether positive or negative. The initial stretch in the school corridor was designed to allow participants to familiarise themselves with the environment and controls. Once in the canteen, and to help participants to navigate the virtual experience, they were provided with a series of instructions, each one directing the participant to approach single avatars or groups of avatars within the canteen (who were further identified with a red diamond over their heads).

There were four brief interactions, each designed to be ambiguous in how they could be perceived and interpreted. Whilst the scenario involves a standardised narrative (i.e. all participants ‘walk’ around the canteen on the same predefined route, and all avatars behave in exactly the same way for all participants), some of these interactions were specifically designed to encourage the participants to explicitly engage with the avatars (e.g. by verbally responding to questions posed by the avatars, or waving hello), thereby to enhance the ecological validity of the paradigm. The four interactions are as follows:

1. Two female students are sitting at a table. As the participant approaches, they ask the participant “We’ve been asked to meet with Miss Fisher. Do you know where her classroom is?”
2. The participant is asked to say hello to Jayden, who is sitting on his own, looking at his phone. When the participant approaches him, Jayden places his bag on the chair next to him, and says “You can’t sit here, I am waiting for someone”.
3. The participant is asked to walk towards and introduce themselves to the group of four students (two girls, two boys) standing by the salad bar. As the participants walk towards them, the students turn to look at the participant and then look away and continue with their conversation.
4. The school bell rings, and the participant is asked to head towards the exit of the canteen. As they do so, a male student walks into the canteen, and as he walks past the participant, waves his hand and says “Heya”.

***State Social Paranoia Scale (SSPS) – shortened version:*** After the walk around the canteen, whilst they are still in their headset, and using the Xbox controller to select their answers, students are asked to answer eight questions to assess levels of paranoid (5 items), neutral (1 item) and positive thoughts (2 items) towards the avatars:

Paranoia items:

Someone was aggressive towards me

Someone was trying to bully me

Someone was trying to frighten me

Someone had it in for me

Someone stared at me in order to upset me

Neutral item:

I felt very safe in their company

Positive items:

Someone wanted to do or say kind things to me

Someone was friendly towards me

***Pilot study:*** Prior to commencing data collection, the virtual reality paradigm went through two iterations of changes following a focus group of 14 young people, and a pilot study with 15 students at one of the participating schools.

***Virtual Reality technical details:*** The virtual paradigm was designed for use in an Oculus Rift head mounted display, which has a resolution of 1080x1200 resolution per eye, a 110 degree field of view, and a 90 Hz refresh rate. The audio for the scene comprised background canteen noises with low-level extracts of conversations, and was delivered on the integrated headphones of the Oculus Rift headset, providing a 3D audio effect.

***Questions administered from the Olweus Bully, Victim Questionnaire [Olweus, 1996]:***

*"The next few questions are about experiences of bullying. Sometimes young people don’t recognise when they are being bullied. Bullying is when someone repeatedly and on purpose says or does mean or hurtful things to another person who has a hard time defending him/herself. How often have the following situations occurred in the past 6 months?*

1. *I was called mean names, was made fun of, or teased in a hurtful way*
2. *Other students left me out of things on purpose, excluded me from their group of friends or completely ignored me*
3. *I was hit, kicked, pushed, shoved around, or locked indoors*
4. *I received mean or hurtful messages, calls or pictures, or other hurtful things via my mobile phone or the Internet”*

Response options were: None; Once or twice; A few times a month; Once a week; A few times a week; Don’t want to answer.

***Questions administered to measure Family (Parent/Sibling) Mental Health Difficulties:***

*Has anyone in your close family (Mum, Dad, and brothers/sisters) experienced a mental health problem?*

Items included: Anxiety; Depression; Bipolar Disorder; Eating disorders (e.g., anorexia or bulimia); Schizophrenia (psychosis); Self-harm; Substance abuse (alcohol / drug problems); Suicide attempt; Other (please specify).

Response options for each of these items were: Yes; No; Don’t know

**Figure S2. Distribution of VR State Paranoid Ideation Scores**

******

| **Table S3.** Sample characteristics of the VR subsample and comparison with full REACH cohort and target population. | | | | | | |
| --- | --- | --- | --- | --- | --- | --- |
|  | | **REACH VR sample**  (n, 481) | | **REACH total sample at T1**  (n, 4353) | | **Target Population* at T1** |
|  | | **n** | **%** | **n** | **%** | **%** |
| **Gender** | |  |  |  |  |  |
|  | Boys | 210 | 43.7 | 2,138 | 49.1 | 50.5 |
|  | Girls | 271 | 56.3 | 2,215 | 50.9 | 49.5 |
| **Free school meals** | |  |  |  |  |  |
|  | No | 385 | 84.4 | 3,137 | 76.3 | 74.8 |
|  | Yes | 52 | 15.6 | 976 | 23.7 | 25.2** |
| **Ethnic group** | |  |  |  |  |  |
|  | Black African | 79 | 16.5 | 1,113 | 25.6 | 27.2 |
|  | Black Caribbean | 33 | 6.9 | 719 | 16.5 | 14.0 |
|  | Mixed | 69 | 14.4 | 617 | 14.2 | 12.5 |
|  | British White | 175 | 36.5 | 667 | 15.3 | 16.4 |
|  | Non-British White | 52 | 10.9 | 626 | 14.4 | 10.8 |
|  | Other | 71 | 14.8 | 1243 | 28.6 | 23.3 |
| **School year** | |  |  |  |  |  |
|  | Year 7 | 92 | 19.0 | - | - | - |
|  | Year 8 | 160 | 33.1 | - | - | - |
|  | Year 9 | 123 | 25.4 | - | - | - |
|  | Year 10 | 98 | 20.2 | - | - | - |
|  | Year 11 | 11 | 2.3 | - | - | - |

T1, Time 1 (2016/17). VR study sample, (2017-2019).

*Lambeth and Southwark Key Stage 3 pupil demographics obtained from the National Pupil Database Spring 2017 School Census.

**Free school meals data for Lambeth and Southwark is not available by Key Stage; the data presented here (% of Lambeth and Southwark pupils receiving free school meals) is for Key Stage 3 and Key Stage 4 pupils combined (25.2%, 2017 Spring Census. Source: Department for Education).

| **Table S4. Impact of bullying at the frequency of once or twice in the last 6 months versus at least twice a month on levels of log-transformed VR paranoid ideation scores** | | | | | | | | | | |
| --- | --- | --- | --- | --- | --- | --- | --- | --- | --- | --- |
|  |  |  | **VR paranoia scores** | | **Unadjusted ^+^** | | **Adjusted ^+^†** | | **Adjusted ^+^**‡ | |
|  | **N** | **%** | **mean** | **s.d.** | **ϐ** | **95 % CI** | **ϐ** | **95 % CI** | **ϐ** | **95 % CI** |
| **Bullying** | | | | |  |  |  |  |  |  |
| None | 185 | 40.0 | 8.76 | 3.26 | - |  | - |  | - |  |
| 1-2 times | 179 | 38.5 | 9.47 | 3.16 | 0.09** | 0.02-0.16 | 0.06 | -0.01; 0.13 | 0.08** | 0.01; 0.15 |
| Monthly + | 99 | 21.4 | 9.95 | 4.09 | 0.12*** | 0.03; 0.20 | 0.08 | -0.01; 0.16 | 0.09** | 0.01; 0.17 |
| ϐ, unstandardised linear regression coefficient; CI, confidence interval; **^+^**accounting for clustering by school; †adjusted for gender, year group, ethnicity, birth place, free school meal status; **p≤0.05; ***p≤0.01 | | | | | | | | | | |

| **Table S5. Associations between bullying and levels of log-transformed VR paranoid ideation scores, adjusting for family mental health problems** | | | | | | | | | | |
| --- | --- | --- | --- | --- | --- | --- | --- | --- | --- | --- |
|  |  |  | **VR paranoia scores** | | **Unadjusted ^+^** | | **Adjusted ^+^†** | | **Adjusted ^+^**‡ | |
|  | **N** | **%** | **mean** | **s.d.** | **Β** | **95 % CI** | **ϐ** | **95 % CI** | **ϐ** | **95 % CI** |
| **Physical bullying** | | | | |  |  |  |  |  |  |
| No | 163 | 51.6 | 8.91 | 3.36 | - |  | - |  | - |  |
| Yes | 153 | 48.4 | 9.56 | 3.64 | 0.06 | -0.02; 0.14 | 0.05 | -0.02; 0.13 | 0.06 | -0.02; 0.13 |
| **Verbal bullying** | | | | |  |  |  |  |  |  |
| No | 225 | 71.4 | 8.95 | 3.34 | - |  | - |  | - |  |
| Yes | 90 | 28.6 | 10.13 | 4.01 | 0.09** | 0.01; 0.18 | 0.06 | -0.02; 0.15 | 0.06 | -0.03; 0.14 |
| **Neglect bullying** | | | | |  |  |  |  |  |  |
| No | 267 | 84.0 | 9.03 | 3.27 | - |  | - |  | - |  |
| Yes | 51 | 16.0 | 10.37 | 4.44 | 0.13** | 0.02; 0.23 | 0.13** | 0.02; 0.23 | 0.12** | 0.01; 0.22 |
| **Cyber bullying** | | | | |  |  |  |  |  |  |
| No | 282 | 88.7 | 9.00 | 3.39 | - |  | - |  | - |  |
| Yes | 36 | 11.3 | 11.47 | 4.29 | 0.24*** | 0.12; 0.36 | 0.23*** | 0.11; 0.35 | 0.23*** | 0.11; 0.35 |
| **Cumulative experiences of bullying** | | | | |  |  |  |  |  |  |
| None | 131 | 41.3 | 8.71 | 3.40 | - |  | - |  | - |  |
| One type | 95 | 30.0 | 9.25 | 3.52 | 0.07 | -0.03; 0.16 | 0.07 | -0.02; 0.16 | 0.07 | -0.02; 0.16 |
| Two types | 47 | 14.8 | 9.34 | 2.83 | 0.06 | -0.06; 0.18 | 0.04 | -0.08; 0.16 | 0.04 | -0.07; 0.16 |
| Three types | 35 | 11.0 | 10.20 | 3.61 | 0.14** | 0.01; 0.28 | 0.13 | 0.01; 0.26 | 0.12 | -0.01; 0.25 |
| Four types | 9 | 2.8 | 13.56 | 6.62 | 0.42*** | 0.18; 0.67 | 0.42*** | 0.18; 0.65 | 0.41*** | 0.17; 0.64 |
| ϐ, unstandardised linear regression coefficient; CI, confidence interval; **^+^**accounting for clustering by school; †adjusted for gender, year group, ethnicity, birth place, free school meal status; ‡adjusted for gender, year group, ethnicity, birth place, free school meal status, family mental health problems; **p≤0.05; ***p≤0.01 | | | | | | | | | | |

| **Table S6. Associations between individual lifetime events and difficulties, and levels of log-transformed VR paranoid ideation scores** | | | | | | | | |
| --- | --- | --- | --- | --- | --- | --- | --- | --- |
|  |  |  | **VR paranoia scores** | | **Unadjusted ^+^** | | **Adjusted ^+^†** | |
|  | **n** | **%** | **mean** | **s.d.** | **ϐ** | **95 % CI** | **ϐ** | **95 % CI** |
| **INTERPERSONAL EVENTS** | | | | | | |  | |
| **Victim of a mugging or robbery ^a^** | | | | |  |  |  |  |
| No | 366 | 84·9 | 9·05 | 3·36 | - |  | - |  |
| Yes | 65 | 15·1 | 10·29 | 3·77 | 0·13*** | 0·04; 0·22 | 0·11** | 0·02; 0·20 |
| **Family member victim of a mugging or burglary ^b^** | | | | |  |  |  |  |
| No | 288 | 66·7 | 8·82 | 3·18 | - |  | - |  |
| Yes | 144 | 33.3 | 10.02 | 3.73 | 0.11*** | 0.05; 0.18 | 0.11*** | 0.04; 0.18 |
| **Physically hit and hurt** **^c^** | | | | |  |  |  |  |
| No | 362 | 85.4 | 9.11 | 3.25 | - |  | - |  |
| Yes | 62 | 14.6 | 9.90 | 4.35 | 0.05 | -0.04; 0.14 | 0.12** | 0.02; 0.22 |
| **Victim of other crime ^c^** | | | | |  |  |  |  |
| No | 405 | 95.5 | 9.13 | 3.30 | - |  | - |  |
| Yes | 19 | 4.5 | 10.95 | 4.77 | 0.15 | -0.01; 0.30 | 0.17** | 0.01; 0.32 |
| **Ethnic or religious discrimination ^d^** | | | | |  |  |  |  |
| No | 266 | 60.2 | 8.92 | 3.06 | - |  | - |  |
| Yes | 176 | 39.8 | 9.65 | 3.90 | 0.08** | 0.01; 0.14 | 0.10*** | 0.03; 0.16 |
| **Self-report ‘Other’ interpersonal events ^e^** | | | | |  |  |  |  |
| No | 387 | 97.0 | 9.09 | 3.31 | - |  | - |  |
| Yes | 12 | 3.0 | 10.83 | 4.78 | 0.12 | -0.06; 0.31 | 0.15 | -0.04; 0.35 |
| **NON-INTERPERSONAL EVENTS** | | | | | | |  | |
| **Had a serious accident ^f^** | | | | |  |  |  |  |
| No | 366 | 83.8 | 9.20 | 3.45 | - |  | - |  |
| Yes | 71 | 16.3 | 9.55 | 3.53 | 0.04 | -0.05; 0.12 | 0.03 | -0.06; 0.11 |
| **Had a serious illness, injury or operation ^d^** | | | | |  |  |  |  |
| No | 267 | 60.4 | 8.90 | 3.31 | - |  | - |  |
| Yes | 175 | 39.6 | 9.80 | 3.59 | 0.10*** | 0.03; 0.16 | 0.13*** | 0.06; 0.19 |
| **Parent or sibling had a serious accident ^g^** | | | | |  |  |  |  |
| No | 338 | 77.0 | 9.11 | 3.37 | - |  | - |  |
| Yes | 101 | 23.0 | 9.74 | 3.60 | 0.07 | -0.01; 0.14 | 0.07 | -0.01; 0.15 |
| **Parent or sibling had a serious illness, injury or accident ^h^** | | | | | | |  |  |
| No | 176 | 39.2 | 8.90 | 2.90 | - |  | - |  |
| Yes | 273 | 60.8 | 9.45 | 3.67 | 0.05 | -0.01; 0.12 | 0.05 | -0.02; 0.11 |
| **Death of a parent or sibling ^i^** | | | | |  |  |  |  |
| No | 397 | 92.5 | 9.28 | 3.46 | - |  | - |  |
| Yes | 32 | 7.5 | 8.72 | 3.21 | -0.04 | -0.16; 0.08 | -0.04 | -0.16; 0.09 |
| **Death of a close family member or friend ^j^** | | | | |  |  |  |  |
| No | 234 | 53.2 | 9.05 | 3.03 | - |  | - |  |
| Yes | 206 | 46.8 | 9.59 | 3.95 | 0.05 | -0.01; 0.11 | 0.04 | -0.03; 0.10 |
| **Involved in a fire or natural disaster ^k^** | | | | |  |  |  |  |
| No | 402 | 93.9 | 9.16 | 3.36 | - |  | - |  |
| Yes | 26 | 6.1 | 10.04 | 4.27 | 0.05 | -0.08; 0.18 | 0.03 | -0.12; 0.17 |
| **Moved from another country because parents/ family were fleeing from a difficult life in home country ^l^** | | | | | | | | |
| No | 428 | 96.4 | 9.19 | 3.37 | - |  | - |  |
| Yes | 16 | 3.6 | 10.13 | 3.69 | 0.14 | -0.03; 0.31 | 0.01 | -0.19; 0.20 |
| **Went to more than one primary school ^m^** | | | | |  |  |  |  |
| No | 315 | 69.4 | 9.25 | 3.28 | - |  | - |  |
| Yes | 139 | 30.6 | 9.27 | 3.74 | 0.01 | -0.06; 0.07 | 0.01 | -0.07; 0.07 |
| **Went to more than one secondary school ^n^** | | | | |  |  |  |  |
| No | 409 | 94.5 | 9.28 | 3.45 | - |  | - |  |
| Yes | 24 | 5.5 | 8.63 | 2.83 | -0.02 | -0.16; 0.11 | -0.01 | -0.15; 0.14 |
| **Temporarily excluded from school ^n^** | | | | |  |  |  |  |
| No | 392 | 90.1 | 9.27 | 3.41 | - |  | - |  |
| Yes | 42 | 9.9 | 9.19 | 3.89 | 0.03 | -0.08; 0.14 | 0.06 | -0.05; 0.18 |
| **Permanently excluded from school ^b^** | | | | |  |  |  |  |
| No | 428 | 99.1 | 9.23 | 3.45 | - |  | - |  |
| Yes | 4 | 0.9 | 10.75 | 3.59 | 0.24 | -0.09; 0.58 | 0.26 | -0.06; 0.59 |
| **Moved home ^o^** | | | | |  |  |  |  |
| No | 163 | 36.1 | 9.12 | 3.45 | - |  | - |  |
| Yes | 288 | 63.9 | 9.39 | 3.49 | 0.03 | -0.03; 0.10 | 0.01 | -0.06; 0.07 |
| **Self-report ‘Other’ non-interpersonal events ^e^** | | | | |  |  |  |  |
| No | 387 | 97.0 | 9.10 | 3.39 | - |  | - |  |
| Yes | 12 | 3.0 | 10.25 | 2.70 | 0.13 | -0.06; 0.32 | 0.10 | -0.09; 0.29 |
| **ADVERSE CHILDHOOD EXPERIENCES** | | | | |  | |  | |
| **Been homeless or lived on the street ^p^** | | | | |  |  |  |  |
| No | 419 | 99.1 | 9.21 | 3.42 | - |  | - |  |
| Yes | 4 | 0.9 | 9.25 | 4.03 | 0.02 | -0.31; 0.35 | 0.04 | -0.29; 0.37 |
| **Family has continuing money problems ^q^** | | | | |  |  |  |  |
| No | 346 | 82.4 | 9.07 | 3.27 | - |  | - |  |
| Yes | 74 | 17.6 | 10.12 | 4.03 | 0.12*** | 0.04; 0.20 | 0.07 | -0.02; 0.16 |
| **Parents drank alcohol so often it caused family problems ^p^** | | | | | | |  |  |
| No | 382 | 90.3 | 9.09 | 3.32 | - |  | - |  |
| Yes | 41 | 9.7 | 9.68 | 2.96 | 0.07 | -0.04; 0.17 | -0.01 | -0.11; 0.11 |
| **Parents often fight or argue ^r^** | | | | |  |  |  |  |
| No | 236 | 55.3 | 8.92 | 3.18 | - |  | - |  |
| Yes | 191 | 44.7 | 9.60 | 3.61 | 0.06* | -0.01; 0.13 | 0.04 | -0.02; 0.11 |
| **Parents divorced or separated ^s^** | | | | |  |  |  |  |
| No | 301 | 69.2 | 9.23 | 3.38 | - |  | - |  |
| Yes | 134 | 30.8 | 9.07 | 3.41 | 0.01 | -0.06; 0.08 | 0.01 | -0.07; 0.08 |
| **In care/foster home/children’s home ^t^** | | | | |  |  |  |  |
| No | 422 | 98.1 | 9.24 | 3.45 | - |  | - |  |
| Yes | 8 | 1.9 | 8.13 | 2.36 | -0.09 | -0.32; 0.15 | -0.11 | -0.34; 0.13 |
| **Self-report ‘Other’ adverse experiences ^e^** | | | | |  |  |  |  |
| No | 374 | 93.7 | 9.14 | 3.42 | - |  | - |  |
| Yes | 25 | 6.3 | 9.08 | 2.61 | -0.04 | -0.18; 0.09 | -0.03 | -0.17; 0.11 |
| ϐ, unstandardised linear regression coefficient; CI, confidence interval; **^+^**accounting for clustering by school; †adjusted for gender, year group, ethnicity, birth place, free school meal status. Missing data for: ^a^50 students; ^b^49 students; ^c^57 students; ^d^39 students; ^e^82 students; ^f^44 students; ^g^42 students; ^h^32 students; ^i^52 students; ^j^41 students; ^k^53 students; ^l^37 students; ^m^37 students; ^n^48 students; ^o^30 students; ^p^58 students; ^q^61 students; ^r^54 students; ^s^46 students; ^t^51 students;  **p≤0.05; ***p≤0.01 | | | | | | | | |

| **Table S7. Associations between life events and difficulties, and levels of log-transformed VR paranoid ideation scores, adjusting for family mental health problems** | | | | | | | | | | |
| --- | --- | --- | --- | --- | --- | --- | --- | --- | --- | --- |
|  |  |  | **VR paranoia scores** | | **Unadjusted ^+^** | | **Adjusted ^+^†** | | **Adjusted ^+^**‡ | |
|  | **n** | **%** | **mean** | **s.d.** | **ϐ** | **95 % CI** | **ϐ** | **95 % CI** | **ϐ** | **95 % CI** |
| **Interpersonal events** | | | | |  |  |  |  |  |  |
| 0 types of events | 113 | 35.3 | 8.29 | 2.78 | - |  | - |  | - |  |
| 1 types of events | 124 | 38.8 | 9.46 | 3.58 | 0.14*** | 0.05; 0.23 | 0.16*** | 0.07; 0.24 | 0.15*** | 0.06; 0.24 |
| 2 types of events | 50 | 15.6 | 9.94 | 3.94 | 0.19*** | 0.07; 0.30 | 0.18*** | 0.06; 0.29 | 0.17*** | 0.05; 0.28 |
| 3+ types of events | 33 | 10.3 | 10.79 | 4.67 | 0.26*** | 0.12; 0.39 | 0.27*** | 0.13; 0.40 | 0.25*** | 0.12; 0.39 |
| **Non-interpersonal events** | | | | |  |  |  |  |  |  |
| 0 types of events | 12 | 3.7 | 8.50 | 2.65 | - |  | - |  | - |  |
| 1 types of events | 60 | 18.6 | 8.43 | 3.18 | -0.06 | -0.28; 0.16 | -0.04 | -0.26; 0.17 | -0.06 | -0.27; 0.16 |
| 2 types of events | 69 | 18.6 | 8.93 | 2.71 | 0.04 | -0.18; 0.26 | 0.04 | -0.17; 0.26 | 0.02 | -0.19; 0.24 |
| 3+ types of events | 190 | 59.0 | 9.68 | 3.96 | 0.10 | -0.11; 0.31 | 0.10 | -0.10; 0.31 | 0.08 | -0.12; 0.29 |
| **Adverse childhood circumstances** | | | | |  |  |  |  |  |  |
| 0 types of events | 132 | 41.6 | 8.89 | 3.32 | - |  | - |  | - |  |
| 1 types of events | 87 | 27.4 | 9.31 | 3.81 | 0.03 | -0.07; 0.13 | 0.03 | -0.07; 0.13 | 0.02 | -0.08; 0.12 |
| 2 types of events | 59 | 18.6 | 10.00 | 4.11 | 0.11** | 0.01; 0.22 | 0.12** | 0.01; 0.23 | 0.09 | -0.02; 0.21 |
| 3+ types of events | 39 | 12.3 | 9.64 | 3.40 | 0.05 | -0.07; 0.18 | 0.02 | -0.11; 0.15 | -0.02 | -0.16; 0.12 |
| ϐ, unstandardised linear regression coefficient; CI, confidence interval; **^+^**accounting for clustering by school; †adjusted for gender, year group, ethnicity, birth place, free school meal status; ‡adjusted for gender, year group, ethnicity, birth place, free school meal status, family mental health problems; **p≤0.05; ***p≤0.01 | | | | | | | | | | |

| **Table S8. Associations between bullying in the previous six months, and lifetime events and difficulties, and highest quartile of VR paranoid ideation scores (n, 102; 21.2% of the sample)** | | | | | | | | | | | | | |
| --- | --- | --- | --- | --- | --- | --- | --- | --- | --- | --- | --- | --- | --- |
|  | **Low paranoia** | | **High paranoia** | | **Unadjusted** | | | **Unadjusted *** | | | **Adjusted *‡** | | |
|  | **n** | **%** | **n** | **%** | **OR** | **95 % CI** | **p** | **OR** | **95 % CI** | **p** | **OR** | **95 % CI** | **p** |
| **Physical bullying** | | | | |  |  |  |  |  |  |  |  |  |
| No | 189 | 81.5 | 43 | 18.5 | - |  |  | - |  |  | - |  |  |
| Yes | 174 | 78.0 | 49 | 22.0 | 1.24 | 0.78; 1.96 | 0.362 | 1.20 | 0.72-2.01 | 0.484 | 1.31 | 0.76; 2.28 | 0.332 |
| **Verbal bullying** | | | | |  |  |  |  |  |  |  |  |  |
| No | 256 | 82.1 | 56 | 17.9 | - |  |  | - |  |  | - |  |  |
| Yes | 108 | 74.5 | 37 | 25.5 | 1.57 | 0.98; 2.51 | 0.063 | 1.39 | 0.81; 2.39 | 0•229 | 1.44 | 0.58; 1.91 | 0.871 |
| **Neglect bullying** | | | | |  |  |  |  |  |  |  |  |  |
| No | 305 | 80.1 | 76 | 19.9 | - |  |  | - |  |  | - |  |  |
| Yes | 60 | 76.0 | 19 | 24.1 | 1.27 | 0.72; 2.26 | 0.413 | 1.22 | 0.64; 2.31 | 0.540 | 1.72 | 0.85; 3.46 | 0.129 |
| **Cyber bullying** | | | | |  |  |  |  |  |  |  |  |  |
| No | 329 | 82.3 | 71 | 17.8 | - |  |  | - |  |  | - |  |  |
| Yes | 37 | 60.7 | 24. | 39.3 | 3.01 | 1.69; 5.34 | <0.001 | 2.79 | 1.46; 5.30 | 0.002 | 3.11 | 1.56; 6.21 | 0.001 |
| **Cumulative experiences of bullying** | | | | |  |  |  |  |  |  |  |  |  |
| None | 152 | 83.5 | 30 | 16.5 | - |  |  | - |  |  | - |  |  |
| One type | 104 | 77.6 | 30 | 22.4 | 1.46 | 0.83; 2.57 | 0.187 | 1.68 | 0.90; 3.16 | 0.106 | 1.94 | 0.99; 3.81 | 0.053 |
| Two types | 59 | 81.9 | 13 | 18.1 | 1.12 | 0.55; 2.29 | 0.763 | 1.14 | 0.51; 2.52 | 0.754 | 1.29 | 0.54; 3.09 | 0.561 |
| Three types | 39 | 72.2 | 15 | 27.8 | 1.95 | 0.96; 3.97 | 0.067 | 1.60 | 0.71; 3.62 | 0.258 | 2.04 | 0.85; 4.91 | 0.112 |
| Four types | 10 | 58.8 | 7 | 41.2 | 3.55 | 1.25; 10.06 | 0.017 | 4.72 | 1.28; 17.36 | 0.020 | 6.90 | 1.67; 28.44 | 0.008 |
| **Interpersonal events** | | | | |  |  |  |  |  |  |  |  |  |
| 0 types of events | 133 | 86.9 | 20 | 13.1 | - |  |  | - |  |  | - |  |  |
| 1 types of events | 145 | 78.4 | 40 | 21.6 | 1.83 | 1.02; 3.30 | 0.042 | 2.15 | 1.10; 4.23 | 0.026 | 2.25 | 1.10; 4.58 | 0.026 |
| 2 types of events | 58 | 77.3 | 17 | 22.7 | 1.95 | 0.95; 3.99 | 0.068 | 2.21 | 0.98; 5.01 | 0.057 | 2.30 | 0.98; 5.42 | 0.056 |
| 3+ types of events | 27 | 62.8 | 16 | 37.2 | 3.94 | 1.81; 8.57 | 0.001 | 4.21 | 1.76; 10.05 | 0.001 | 4.77 | 1.84; 12.39 | 0.001 |
| **Non-interpersonal events** | | | | |  |  |  |  |  |  |  |  |  |
| 0 types of events | 21 | 77.8 | 6 | 22.2 | - |  |  | - |  |  | - |  |  |
| 1 types of events | 74 | 85.1 | 13 | 14.9 | 0.61 | 0.21; 1.81 | 0.378 | 0.86 | 0.23; 3.16 | 0.816 | 0.75 | 0.19; 2.90 | 0.676 |
| 2 types of events | 70 | 81.4 | 16 | 18.6 | 0.80 | 0.28; 2.30 | 0.679 | 0.82 | 0.22; 3.04 | 0.773 | 0.79 | 0.20; 3.06 | 0.733 |
| 3+ types of events | 206 | 76.6 | 63 | 23.4 | 1.07 | 0.41; 2.77 | 0.888 | 1.43 | 0.44; 4.71 | 0.548 | 1.28 | 0.38; 4.33 | 0.694 |
| **Adverse childhood circumstances** | | | | |  |  |  |  |  |  |  |  |  |
| 0 types of events | 148 | 80.4 | 36 | 19.6 | - |  |  | - |  |  | - |  |  |
| 1 types of events | 107 | 78.1 | 30 | 21.9 | 1.15 | 0.67; 1.99 | 0.609 | 1.33 | 0.72; 2.46 | 0.355 | 1.39 | 0.73; 2.66 | 0.319 |
| 2 types of events | 63 | 77.9 | 18 | 22.2 | 1.17 | 0.62; 2.22 | 0.621 | 1.40 | 0.69; 2.83 | 0.354 | 1.33 | 0.62; 2.83 | 0.463 |
| 3+ types of events | 41 | 78.9 | 11 | 21.2 | 1.10 | 0.52; 2.36 | 0.800 | 1.06 | 0.46; 2.43 | 0.898 | 0.61 | 0.24; 1.56 | 0.297 |
| OR, Odds Ratio; CI, confidence interval; *accounting for clustering by school; ‡ adjusted for gender, year group, ethnicity, birth place, free school meal status; | | | | | | | | | | | | | |
